# Utilization Patterns and Projected Demand of Antiretroviral Drugs in Low- and Middle-Income Countries

**DOI:** 10.1155/2011/749041

**Published:** 2011-03-24

**Authors:** Françoise Renaud-Théry, Carlos Avila-Figueroa, John Stover, Sigrid Thierry, Marco Vitoria, Vincent Habiyambere, Yves Souteyrand

**Affiliations:** ^1^Department of HIV/AIDS, World Health Organization, 1211 Geneva, Switzerland; ^2^AIDS Financing and Economics Division, UNAIDS, Geneva, Switzerland; ^3^Futures Institute, Glastonbury, CT 06033, USA

## Abstract

*Background*. The rapid scale-up of antiretroviral therapy in resource-limited settings has greatly increased demand for antiretroviral medicines and raised the importance of good forward planning, especially in the context of the new 2010 WHO treatment guidelines. *Methods*. Forecasting of the number of people receiving antiretroviral therapy from 2010 to 2012 was produced using three approaches: linear projection, country-set targets, and a restricted scenario. Two additional scenarios were then used to project the demand for various antiretroviral medicines under a fast and slower phase-out of stavudine. *Results*. We projected that between 7.1 million and 8.4 million people would be receiving ART by the end of 2012. Of these, 6.6% will be on second-line therapy. High variation in forecast includes reductions in the demand for d4T and d4T increases in the demand for tenofovir, emtricitabine followed by efavirenz, ritonavir, zidovudine and lopinavir; lamivudine, atazanavir, and nevirapine. *Conclusion*. Despite the global economic crisis and in response to the revised treatment guidelines, our model forecasts an increasing and shifting demand for antiretrovirals in resource-limited settings not only to provide treatment to new patients, but also to those switching to less toxic regimens.

## 1. Introduction

In the past five years, low- and middle-income countries have aggressively scaled up HIV treatment. By the end of 2009, more than 5.2 million people were receiving antiretroviral therapy [1]. This represented an increase of more than 1.2 million people from the end of 2008 and a 13-fold expansion over the previous six years. This rapid scale-up has greatly increased the demand on antiretroviral medicines and raised the importance of good forward planning.

The new forecasts presented here update previous estimates [2–4] by adding updated information on the number of people receiving antiretroviral therapy and data from the annual WHO AIDS Medicines and Diagnostics Service (AMDS) survey on the use of antiretrovirals [5–7]. Two major elements taken into account for developing updated forecasts were the financial crisis and the progressive implementation of the new WHO antiretroviral therapy (ART) recommendations published in 2010 [8].

Under the revised WHO guidelines, an estimated 14.6 million (13.4–15.4 million) people in low- and middle-income countries will be eligible for ART, a 45% increase from the 10.1 million (9.0–11.1 million) people in need of ART under the previous guidelines. While the predicted expansion of HIV treatment is well below 14.6 million, the figures in this paper are not a statement about what should be or will be accomplished in 2012. Rather, these estimates indicate what is likely to be accomplished in the absence of meaningful changes in the conditions driving treatment scale-up.

## 2. Methods

Estimates of the number of people receiving antiretroviral therapy were forecast from 2010 to 2012 using three approaches: linear projection, country-set targets and restricted scenario. To forecast the demand for antiretroviral medicines, two additional scenarios were included: a fast and slower phase-out of stavudine, with a baseline consumption taken from the 2009 WHO survey on antiretroviral use. Once the number of patients on treatment was estimated, these numbers were translated into volumes of active pharmaceutical ingredients.

### 2.1. Multicountry Survey on ARV Use

In March 2009, the WHO Department of HIV and AIDS conducted the third annual survey to assess antiretroviral use in low- and middle-income countries. A standard questionnaire was sent to the 43 countries with the highest number of people receiving antiretroviral therapy as of December 2008. National AIDS programmes of 39 countries responded and completed the questionnaires; these were collected by the WHO countries and regional offices and forwarded to the WHO AMDS team in Geneva.

### 2.2. Forecasting the Number of Patients Receiving Treatment

#### 2.2.1. Choice of Forecasting Approach

Three forecast approaches were considered: linear projection, country-set targets, and restricted scenario. The linear projection is based on projections of historical trends of the number of people receiving antiretroviral therapy by country, from the data reported between December 2006 and December 2008 for each country. This approach is a linear extrapolation using a regression line through the last three data points to determine the average annual increase. This increase is added to the estimated number of people receiving antiretroviral therapy as of December 2008. This approach has the advantage of being grounded in actual country-observed data and is easy to implement and understand. Since it could result in a projection greater than total estimated need, all projections were capped at the level established for the country-set target scenario. Recent growth has been approximately linear, so this approach represents the best projection, assuming that past trends continue.

The second scenario was adjusted using targets set by countries for the number of people that they expect to reach with antiretroviral therapy by 2012. These targets take into account the realities in each country and their goals for increasing coverage. During a consultation at the WHO in Geneva in October 2009, five countries reported their targets: India, Kenya, South Africa, Thailand, and Zambia. Together, these countries account for approximately 40% of people receiving antiretroviral therapy. In the country-set target projection, it is assumed that the total number of people receiving antiretroviral therapy will grow at the same rate as the projected number for the five countries.

The two scenarios using linear projection and country targets may be over-optimistic if the economic crisis results in slower growth in international and national financing of antiretroviral therapy programmes [9–11]. A restricted scenario was therefore created to consider this possibility. Under the restricted scenario, the number of people receiving antiretroviral therapy grows at only 75% of the annual rate of the target scenario.

The three methods described above were applied to each of the 154 countries in the analysis and the results were aggregated to regional and interregional totals.

This exercise defined the number of people receiving antiretroviral therapy as the number of people receiving antiretroviral therapy at the end of a given year. This includes people who started antiretroviral therapy in that year, as well as those who started in previous years and remained on treatment. 

#### 2.2.2. Retention Assumptions

The proportion of people receiving antiretroviral therapy in one year who continue on antiretroviral therapy through the same programme in a subsequent year is called the retention rate. A number of countries estimate retention rates from their programme data. Published data from the *Towards Universal Access 2009* report have been consolidated into first year and subsequent years, resulting in a 79.5% overall retention rate for the first year and 95.8% for subsequent years [5].

#### 2.2.3. First- and Second-Line Antiretroviral Therapy

The current proportion of people receiving second-line therapy was extracted from the 2009 WHO survey on antiretroviral use. The number of people receiving first-line antiretroviral therapy in any year is projected as the number of people initiating antiretroviral therapy in that year plus those people receiving first-line therapy in the previous year who survive to this year and do not switch to second-line therapy. For countries without information on the number of people receiving second-line therapy, a regional average from the survey was applied: 2.8% for sub-Saharan Africa; 8.2% for Central America and the Caribbean; 9.1% for Eastern and Central Europe; 30% for Latin America; 0.2% for South-East Asia; 2.0% for Western Pacific.

#### 2.2.4. Rate of Switching from First- to Second-Line Antiretroviral Therapy

Data from a systematic review of monitoring strategies, treatment failure, and attrition rates, conducted by WHO in collaboration with the Australian National Centre in HIV Epidemiology and Clinical Research, were used to estimate the regional failure rates [12]. The review showed that when viral load monitoring is used, a failure rate of 6% is detected and that when the CD4 count is used there is a 1.9% detected failure rate. Data from the review were used to estimate failure rates for Africa (2.6%), Latin America (2.6%), and Asia (1.1%). For countries in all other regions, the average of 1.9% was used. For Latin American countries where viral load is used routinely, including Argentina, Brazil, Mexico, and Venezuela, the 6% rate from the meta-analysis was used.

### 2.3. Forecasting Demand for Antiretroviral Medicines

One of the expected impacts of the new WHO recommendations on the use of antiretroviral drugs in low- and middle-income countries will be a progressive phasing out of stavudine and an increase in first-line regimens based on zidovudine and tenofovir [8]. The antiretroviral demand forecasts presented are based on two scenarios, which consider either a fast or slow phase-out of stavudine. The two scenarios were developed for both first- and second-line regimens, and the population was divided into two categories within each scenario: people already receiving antiretroviral therapy and people initiating antiretroviral therapy, for whom the new recommendations apply differently. The baseline is taken from the 2009 WHO AMDS survey on antiretroviral use.

## 3. Results

### 3.1. Use of Antiretroviral Therapy in Low- and Middle-Income Countries as of December 2008

Thirty-nine national AIDS programmes responded to the survey, representing a total of 3.4 million people receiving antiretroviral therapy, or about 85% of the estimated 4.0 million people receiving antiretroviral therapy in resource-limited countries as of December 2008. In these 39 countries, 93% of the people receiving antiretroviral therapy were adults (3.2 million adults) and 7% were children (252 000 children). 

An exploratory data analysis confirmed that the pattern of use of first- and second-line regimens was similar among the 37 countries that scaled up treatment programmes after the publication of WHO's public health approach to antiretroviral therapy in 2002 [13]. However, in Brazil and Mexico, where the expansion of treatment programmes started earlier, the pattern of antiretroviral use was significantly different, particularly for the level of use of second-line regimens. For this reason, data from Brazil and Mexico were analysed separately from data from other programmes that have scaled up more recently and are presented separately.

**Figure 1:**
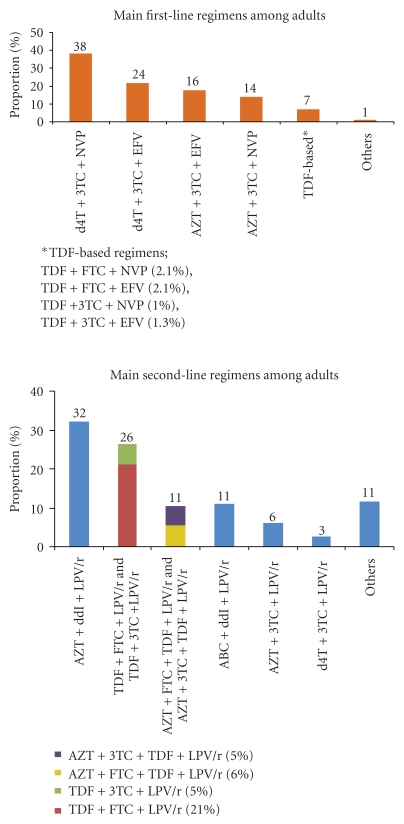
First- and second-line antiretroviral regimens received by adults in 37 low- and middle-income countries (*n* = 2 870 000 and *n* = 67,500), December 2008.

Figure 1 shows the composition and distribution of the first- and second-line regimens most commonly used in the 37 low- and middle-income countries. In antiretroviral treatment programmes, the vast majority of adults (98%) were receiving first-line regimens and 2% of patients were receiving second-line regimens. A vast majority (99%) of people on first-line ART, and 87% of those on second-line ART, were receiving regimens recommended by the WHO [14]. More than half of the patients on first-line regimens (62%) were receiving stavudine and 56% were receiving nevirapine as the nonnucleoside component. Among the 2% of patients receiving second-line regimens, a majority (55%) were on a regimen containing zidovudine and 43% were on one containing tenofovir. Lopinavir boosted with low-dose ritonavir (LPV/r) was the predominant protease inhibitor, received by 95% of adults. A vast majority of children (97%) were receiving first-line regimens (237 000 children), with an almost equal distribution between regimens containing stavudine and zidovudine (49% and 47%, resp.). Brazil and Mexico reported frequent use of second-line regimens in adults (20% and 13%, resp.) and very low use of stavudine and nevirapine in first-line regimens. A high proportion of children in Brazil and Mexico were receiving second-line therapy (40% and 13%, resp.).

### 3.2. Antiretroviral Market Trends in 17 Countries from 2006 to 2008

An analysis of 17 countries (Burkina Faso, Burundi, Cambodia, Cameroon, Côte d'Ivoire, Ethiopia, India, Kenya, Lesotho, Namibia, Nigeria, Rwanda, Swaziland, the United Republic of Tanzania, Uganda, Zambia, and Zimbabwe.) that responded to all three rounds of the WHO survey on ARV use (2006–2008) showed the antiretroviral market trends over the three-year period [5]. The analysis confirmed that the proportion of adults receiving second-line regimens in antiretroviral therapy programmes remains low with 2.3% in 2008 (3.5% in 2007 and 2% in 2006). There was a net increase in tenofovir use in 2008, with 9% and 56% of adults receiving tenofovir-based regimens on first and second-line respectively. (1.5% for first line and 32% for second line in 2007, and <1% and 11% in 2006). The analysis also showed a slow decrease in stavudine use, with 57% of adults on first-line regimens receiving stavudine in the 17 countries in 2008 (68% in 2007 and 67% in 2006) and an increase in zidovudine use, with 35% of adults on first-line regimens receiving zidovudine in 2008 (26% in 2007 and 29% in 2006).

### 3.3. Evolution in National Antiretroviral Therapy Guidelines

The survey showed that WHO 2006 treatment recommendations have largely been adopted and implemented by national HIV programmes, despite a few people still receiving treatment regimens that are not in line with WHO guidelines (such as second-line regimens without a protease inhibitor backbone) [14]. There is a constant evolution in national treatment guidelines; half the countries were in the process of revising adult (16 countries) and paediatric (19 countries) protocols at the time of the survey. The most frequently planned changes in adult guidelines were the introduction of tenofovir (nine countries), a change from stavudine as the preferred first-line option (seven countries), an increase in the CD4 threshold for treatment initiation to below 250 cells/mm^3^ (two countries) or below 350 cells/mm^3^ (four countries), and the introduction of viral load testing to monitor treatment (three countries).

### 3.4. Forecast Numbers of People Receiving Antiretroviral Therapy in Low- and Middle-Income Countries by 2012

**Figure 2:**
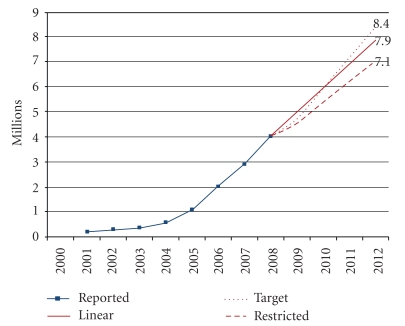
Number of people receiving antiretroviral therapy in low- and middle-income countries, reported on December 2008 and projected for December 2009–December 2012, linear projection and two scenarios (millions).

Figure 2 shows the forecast demand for ART, based on linear projections, country-set targets, and the restricted scenario, which projects between 7.1 and 8.4 million people receiving ART in low- and middle-income countries by the end of 2012. The baseline number of people receiving ART was 4.0 million by the end of 2008 and is projected to increase to 5.0 million by the end of 2009, 6.0 million by the end of 2010, 6.9 million by the end of 2011, and 7.9 million by the end of 2012, according to linear projection. Annual growth is slightly slower than the growth observed between 2007 and 2008. It is expected that countries with the greatest number of people receiving antiretroviral therapy would experience slower enrolment of new patients, after achieving high coverage levels. When using upper and lower estimates of the number of people receiving antiretroviral therapy between 2006 and 2008 in each country, the estimated annual increase ranged from 0.98–1.0 million people per year. The country-set target scenario forecasts lower numbers than the linear projection in 2009 and 2010, but greater numbers by 2012, reaching a total of 8.4 million (6.3% greater than the linear projection). The restricted scenario forecasts the lowest numbers receiving antiretroviral therapy, reaching only 7.1 million by 2012 (10% less than the linear projection).

Table 1 shows detailed results for the linear projection approach, where the number of people receiving second-line therapy is estimated to increase from around 190 000 in 2008 to 520 000 in 2012, equivalent to an increase from 4.8% to 6.6%. Under the country-set target scenario, the number of people receiving second-line therapy is estimated to increase from about 190 000 in 2008 to 530 000 in 2012, a relative increase from 4.8% to 6.4%. Under the restricted scenario, the number of people receiving second-line therapy is estimated to increase from about 190 000 in 2008 to 500 000 in 2012, or from 4.8% to 7.1%.

### 3.5. Forecasting Demand for Antiretroviral Medicines

Table 2 presents the resulting forecast quantities of antiretroviral products required globally, using the linear projection approach. The greatest variation in forecast demand is for stavudine, which ranges from a decrease in demand of 39% (from 2008 to 2012) with fast phase-out, to an increase of 27% with slower phase-out. For other antiretroviral molecules, the greatest annual increases are forecast for tenofovir and emtricitabine; followed by efavirenz, ritonavir, zidovudine, and lopinavir; lamivudine, atazanavir, and nevirapine.

## 4. Discussion

We forecast that an estimated 7.9 million people will be receiving antiretroviral therapy in low- and middle-income countries by 2012, based on the linear projection model. Taking into account the strengths and weaknesses of each method, the results of the linear projection are being used as the main basis for analysis and forecasting. Recent growth has been approximately linear and may be the best approach to extrapolate past trends over short periods of time. The other two scenarios are included to show the range of uncertainty around the projections (7.1–8.4 million).

An important strength of these projections is the inclusion of two unique sources of data: the latest WHO annual survey on antiretroviral use and a country target-setting consultation on future antiretroviral therapy coverage. Although conservative, these are still optimistic projections, since many countries would require formidable implementation efforts and additional funding to scale up treatment services at the pace observed in the recent past and to continue enrolling an estimated 83,000 new people on ART per month. However, treatment demand and the supply of funding to purchase treatment were found difficult to separate. Demand for ART in low-resource countries where donors provide the majority of ART funding may be more subject to the availability of funding from donor agencies than domestic constraints.

The projected estimates under the linear model suggest that coverage would grow by 2.9 million people in three years, representing a 60% increase over the number of people receiving ART by the end of 2012. However, this forecast must be viewed within the context of various factors likely to influence future demand: the global economic downturn, the delivery capacity of poor countries with the greatest HIV burden, and the implementation of the new WHO guidelines.

The global financial crisis is threatening countries' ability to sustain the progress made so far with respect to antiretroviral coverage, as well as efforts to attain universal access goals. It is therefore necessary to better understand the effect of the financial crisis on antiretroviral demand, its possible consequences, and what can be done to avoid negative impact. After years of significant increases for international AIDS assistance provided by the G8, European Commission, and other donor governments, funding remained essentially flat over the 2008-2009 period. Disbursements were $7.6 billion in 2009, compared to $7.7 billion in 2008. The observed decrease in disbursements between 2008 and 2009 is difficult to interpret, due to currency fluctuations and reporting cycle [9–11]. The global economic crisis is not the only obstacle and other donor priorities, such as global health, could likely impact the supply of aid for ART.

Some of the countries with the fastest growth in the number of people receiving ART are reaching high levels of coverage and may be nearing a saturation point of what they can deliver with their existing health care infrastructure. In some other cases, a saturation point may also be reached while high coverage remains to be attained. Growth in ART coverage in these countries can therefore be expected to slow down in the near future. The changes in the new WHO recommendations to initiate ART at a threshold of CD4 count of 350 cells/mm^3^ will lower coverage levels in countries, especially those with very high burden of disease. The capacity of the health system to enroll new patients and to sustain patients on ART in the long term will also prove to be a central challenge.

The other factor influencing demand is the standardization of treatment and the application of clinical guidelines. The demand scenarios for antiretroviral medicines are based on the assumption that most low- and middle-income countries will progressively implement the new WHO treatment recommendations. Since the introduction of WHO ART guidelines in 2002 (and their subsequent updates in 2003 and 2006), successive AMDS surveys have shown an increasing rate of country compliance with WHO guidelines. Adoption of the new guidelines involves two key components: the duration of the transition period for countries to fully implement the new recommendations and the level of continued use of stavudine.

Treating the maximum possible number of new patients has been the priority for many governments and donors, resulting in increased demand for less expensive formulations (first-line stavudine-containing regimens procurement price dropped to between $64 and $122) [15]. One of the major recommendations of the 2010 WHO ART guidelines is to provide more durable, efficacious, and tolerable antiretroviral first-line regimens by substituting other options for stavudine-containing regimens. Many countries have already started to change their national guidelines or have entered a transition period to phase out stavudine in favour of zidovudine or tenofovir for people initiating first-line antiretroviral therapy.

A trend comparison across results from the 2006–2008 surveys for 17 countries confirms a decrease in the use of stavudine and an increase in the use of zidovudine and tenofovir. Countries may choose a longer transition period to phase out stavudine in order to reduce the cost burden of scaling up the number of people initiating antiretroviral therapy. Despite substantial reduction over the years, first-line regimens that include tenofovir or zidovudine cost up to three times more than stavudine-based regimens, and prices of second-line regimens are still high. The annual price per patient for first-line regimens ranges from $136 to $243 in low-income countries, and from $116 to $667 in lower-middle-income countries. For second-line regimens, the annual price per patient ranges from $572 to $803 in low-income countries and from $818 to $1545 in lower-middle-income countries. In upper-middle-income countries, annual prices of first-line regimens range from $161 to $1033, while second-line regimens range from $3393 to $3647 [15]. The transition period would most likely depend on the availability of resources over the next three years. Certain countries with large treatment volumes, such as Kenya, Nigeria, and South Africa, will continue to be instrumental in driving demand.

Opportunities for a faster phase-out include new prequalified formulations of fixed-dose combinations, possible further price reductions for medicines, and available funding for antiretroviral treatment programmes. In addition, some countries are already seeking efficiency savings through better procurement of medicines and laboratory tests, to sustain and expand treatment programmes [16].

Given these factors, while we forecast an increasing-linear demand for antiretroviral medicines, its trajectory would more likely occur within the boundaries of the country-set targets and the restricted scenario. While the country-set target scenario uses data provided by the countries themselves, it may be over-optimistic if the economic crisis results in slower growth in international and national financing of antiretroviral therapy programmes. The resulting slower growth is difficult to estimate. If funding restrictions reduce the rate of growth by 25%, as per the restricted scenario, then 800,000 fewer people would be receiving antiretroviral therapy by 2012.

As in any other model, our projections have some limitations. First, the projections are based on a small number of past observation years. Second, while the WHO surveys are broadly representative of ART programmes operating across low- and middle-income countries, their general applicability requires careful consideration. This is especially true when extrapolating information from large country programmes that account for a high number of patients to countries with small-sized programmes operating in a wide variety of conditions. Programme size and other features of ART services that affect scale-up rates require further investigation. Future projections should address the barriers preventing countries from rapid scale-up and the implementation of standard guidelines.

Our results support the notion that the demand of ART in resource-limited settings will keep increasing and that not only will new patients be starting ART, but those in long-term care will also be shifted to less toxic antiretroviral drugs. Although the price of antiretroviral medicines has been the most important factor affecting their demand, government and donors are shifting to less toxic ARVs and bulk purchasing will eventually push prices down. It is expected that the use of more patient-friendly ARVs will be also associated with better retention rates and better overall outcomes [17, 18].

In conclusion, our demand estimates are still below the accelerated coverage required to reach universal access in resource-limited countries. Using classical methods of demand forecasting, we estimated the quantities of both antiretroviral medicines and the number of clients receiving treatment, based on historical demand. We believe this information to be relevant for informed demand planning. These forecasts should not downplay the fact that many people living with HIV would benefit from an accelerated scale-up of ART. This information should be supplemented with projections using different approaches and based on strategic targets. These reports should be made available to donors and governments as part of the continuing effort to provide information about the resources needed to reach universal access to HIV treatment and care.

##  Conflict of Interests

The authors declare no conflict of interest.

## Figures and Tables

**Table 1:** People receiving antiretroviral therapy in low- and middle-income countries, by type of regimen, reported for 2008 and forecast for 2009–2012, linear projection (millions).

	2007	2008	2009	2010	2011	2012
Number on first line (millions)	2.84	3.84	4.74	5.62	6.49	7.35
Percent	95.1%	95.2%	94.8%	94.4%	93.9%	93.4%

Number on second line (millions)	0.15	0.19	0.26	0.34	0.42	0.52
Percent	4.9%	4.8%	5.2%	5.6%	6.1%	6.6%

Total (millions)	2.99	4.03	5.00	5.96	6.92	7.88

**Table 2:** Demand for antiretroviral medicines for the linear projection with fast and slower phase-out of stavudine, 2008–2012 (person-years of antiretroviral therapy) (millions).

	Year												
	2008	2009			2010			2011			2012		

Molecule	Reported^a^	Fast d4T phase-out^a^	Slower d4T phase-out^a^	Difference between scenarios	Fast d4T phase-out^a^	Slower d4T phase-out^a^	Difference between scenarios	Fast d4T phase-out^a^	Slower d4T phase-out^a^	Difference between scenarios	Fast d4T phase-out^a^	Slower d4T phase-out^a^	Difference between scenarios
d4T	1.92	2.12	2.23	5%	2.00	2.41	20%	1.68	2.48	47%	1.18	2.43	105%
ZDV	1.26	1.67	1.60	−4%	2.15	1.92	−11%	2.68	2.23	−17%	3.26	2.56	−22%
3TC	3.23	4.03	4.00	−1%	4.73	4.70	−1%	5.40	5.36	−1%	6.06	5.96	−2%
NVP	2.00	2.34	2.30	−2%	2.70	2.61	−3%	3.00	2.85	−5%	3.26	3.00	−8%
EFV	1.15	1.72	1.72	0%	2.14	2.16	1%	2.59	2.64	2%	3.08	3.14	2%
ABC	0.05	0.06	0.07	12%	0.06	0.09	46%	0.05	0.11	115%	0.04	0.14	297%
ddI	0.08	0.09	0.10	9%	0.09	0.12	35%	0.07	0.14	95%	0.03	0.15	360%
IDV	0.01	0.01	0.01	36%	0.01	0.02	142%	0.01	0.03	345%	0.01	0.05	750%
LPV	0.18	0.22	0.22	0%	0.28	0.28	1%	0.34	0.35	3%	0.41	0.43	4%
TDF	0.21	0.54	0.50	−8%	1.02	0.89	−13%	1.63	1.38	−15%	2.37	1.99	−16%
FTC	0.10	0.25	0.24	−2%	0.38	0.36	−4%	0.55	0.51	−6%	0.74	0.69	−7%
NFV	0.01	0.01	0.01	−6%	0.01	0.01	−20%	0.01	0.00	−45%	0.00	0.00	−100%
RTV	0.16	0.22	0.22	2%	0.28	0.29	6%	0.34	0.38	10%	0.41	0.48	15%
SQV	0.00	0.00	0.00	69%	0.00	0.01	228%	0.00	0.01	448%	0.00	0.02	730%
ATV	0.03	0.03	0.03	8%	0.03	0.04	28%	0.04	0.06	54%	0.04	0.08	88%

^a^Units are millions of person-years of antiretroviral therapy.
